# Co-producing a physical activity intervention in two forensic psychiatry settings in the UK: The IMPACT study

**DOI:** 10.1192/j.eurpsy.2023.930

**Published:** 2023-07-19

**Authors:** T. Walker

**Affiliations:** Psychology, Durham University, Durham, United Kingdom

## Abstract

**Introduction:**

In the UK there are 3500 individuals detained in medium secure forensic psychiatry units. Service users in such settings have complex and serious mental illness (SMI), often with co-morbid physical health problems and a life expectancy of at least 10 years shorter than the general population. They often have low levels of physical activity. There is little evidence about physical activity interventions for medium secure service users in the United Kingdom.

**Objectives:**

Our objective is to co-produce, with medium secure service users, the content and delivery of an intervention to increase physical activity. We shall assess feasibility, acceptability and pilot data collection methods for outcomes relevant for a future randomised controlled trial.

**Methods:**

This is a 24-month mixed-methods project that will follow the Medical Research Council (MRC) framework Developing and Evaluating Complex Interventions. The study has 4 phases.

- Phases 1-2 will gather information required to co-develop an evidence based intervention in Phase 3.

- Phase 4 will assess the intervention in a feasibility study, evaluating and testing the intervention for a future pilot study.

Study settings: Two NHS Medium Secure In-Patient Psychiatric Hospitals in the UK.

**Results:**

This paper presents the findings from the Phase 1 questionnaire and focus groups with service users and hospital staff that identified the barriers and facilitators to physical activity in such settings. The results are then discussed in relation to the Phase 2 qualitative results that explored stakeholders’ and service users’ opinions into how to increase physical activity among medium secure service users by identifying potential elements for inclusion in a physical activity intervention, to gain insight into how we can establish engagement of this group with the intervention, maintain commitment, avoid drop-out and develop the intervention design. All findings are presented using the Capability, Opportunity, and Motivation Model of Behaviour (COM-B model), which is widely used to identify what needs to change for a behaviour change intervention to be effective.

**Conclusions:**

The findings of Phases 1-2 are allowing the team to move forward with Phase 3 that is currently developing an intervention to increase physical activity for adult inpatient service users in the medium secure units. This phase will be guided by the MRC framework and the COM-B model to define the target behaviours and select the most suitable intervention components (functions and techniques) and implementation approach.

**Disclosure of Interest:**

None Declared

